# The Year-Book of American Contributions to Medical Science and Literature

**Published:** 1861-05

**Authors:** 


THE YEAR-BOOK OF AMERICAN CONTRIBUTIONS TO MEDICAL SCIENCE AND LITERATURE.—At the request of Dr. O. C. Gibbs, of Frewsburg, Chautauque County, N. York, we announce that his proposed publication has been delayed on account of the unsettled state of the country, and that so soon as one thousand names have been received the work will be put to press. The subscription price being but three dollars, and the enterprise a worthy one, we trust that the profession will give Dr. Gibbs all necessary encouragement.

